# Kinetics in lumbosacral and lower-limb joints of sprinters during barbell hip thrust compared to deadlift and back squat

**DOI:** 10.1371/journal.pone.0251418

**Published:** 2021-07-01

**Authors:** Mitsuo Otsuka, Toyoyuki Honjo, Akinori Nagano, Tadao Isaka

**Affiliations:** 1 Faculty of Sport Science, Nippon Sport Science University, Tokyo, Japan; 2 Department of Mechanical Systems Engineering, National Defense Academy, Yokosuka, Japan; 3 Faculty of Sport and Health Science, Ritsumeikan University, Kyoto, Japan; Texas State University, UNITED STATES

## Abstract

Joint kinetic characteristics during the eccentric phase are important in resistance exercises because eccentric actions with elastic potential energy storage lead to the energy recoil with large joint moment and power generation during the subsequent concentric phase. Previous studies assessed the force production capacity in the barbell hip thrust; however, these were reported by the methodology using only surface electromyographic amplitudes recorded in the lower back and thigh muscles and did not focus on eccentric action. This study aimed to determine kinetic characteristics of lumbosacral, hip and knee joints of sprinters during the eccentric and concentric phases in a barbell hip thrust, compared to those of deadlift and back squat. Eleven well-trained male sprinters participated in this study. Each participant performed two full ranges of motion repetition using their previously determined six-repetition maximum loads. During strength exercises, reflective marker displacements attached to the body and a barbell were captured using 22 high-speed cameras, and ground reaction forces were captured using 4 force plates simultaneously. In the barbell hip thrust, as well as deadlift, the peak values of the lumbosacral and hip extension moments were generated almost immediately after the eccentric phase and were 24% and 42% larger than those in the back squat, respectively. In the knee joint, the largest was the peak extension moment in the back squat (155 ± 28 Nm), followed in order by that in the barbell hip thrust (66 ± 33 Nm) and that in the deadlift (24 ± 27 Nm). These demonstrated that a barbell hip thrust, as well as deadlift, can be a resistance exercise to strengthen the lower back and posterior thigh muscles. Thus, these resistance exercises may be able to be used separately according to their intended purposes, enabling transformations of strength training to specific dynamic motions such as sprint running.

## Introduction

A barbell hip thrust is regarded as a representative hip extension exercise in resistance training [1–4], where the gluteus maximus is substantially activated [5–8]. Therefore, the barbell hip thrust is a useful resistance exercise for athletes, such as sprinters, who often activate the hip extensor muscles [9–11].

Previous studies explained the force production capacity in the barbell hip thrust and compared that to other exercises. These were reported by the methodology using only surface electromyographic (EMG) amplitudes recorded in the lower back and thigh muscles [6,8]. For example, a deadlift is clearly superior for activating the biceps femoris than a barbell hip thrust [5], suggesting that the hip extension strength is probably lower in the barbell hip thrust than that in the deadlift. However, the EMG amplitude evaluates only the number of active motor units in a muscle. A muscle force also changes by the joint angular displacement due to a force-length relationship in a muscle [12]. Moreover, the joint moment cannot increase when the moment arm, which is a vector perpendicular to the muscle tendon complex from the joint center, is shortened by over-flexion/extension in the joint. So, many previous studies showed that EMG amplitudes were not fully associated with the magnitude of force generated during the isokinetic test [13,14]. Therefore, comparing the joint kinetic parameters involving joint moments is required between the barbell hip thrust and the other exercises so as to explain the detailed extension strength. However, to the best of our knowledge, there were no peer-reviewed papers on the joint kinetics in the barbell hip thrust.

Moreover, the EMG studies on the barbell hip thrust are limited to analyzing the amplitudes only during the isometric [6] or concentric phase [5,7,15]. In both high- and low-load resistance trainings, eccentric actions with elastic potential energy storage lead to recoil with high joint moment and power generation during the subsequent concentric phase [16,17]. This action of the muscle-tendon complex is called a stretch-shortening cycle [18,19]. When compared to training focused only on concentric action, training focusing on eccentric action can efficiently increase the muscle stiffness through mechanical adaptation and finally improve dynamic lower-limb motions with the stretch-shortening cycle [20,21]. Similar transformation of strength training into specific dynamic motions is observed in conventional lower-limb resistance training such as squat or deadlift [10,22], which is probably caused by involvement of eccentric load. The power output capacity with the stretch-shortening cycle is a crucial factor in lower limbs for many dynamic motions involving sprint running [23–26], jumping [27], cycling [28], swinging [29] and so on. Therefore, in the barbell hip thrust, investigating joint kinetic characteristics during the eccentric phase is important to supplement knowledges of concentric actions by the previous studies [5,6,8]. This ultimately would lead to discuss the similarity and difference between kinetics in the barbell hip thrust and that in dynamic motions.

It has been reported that barbell hip thrust training can maximize many dynamic motions involving sprint runs and jumps [10]. In particular, sprint running performance can be chronically enhanced by barbell hip thrust training compared to squat training, which is a representative standing exercise [10]. The higher sensitivity of barbell hip thrust training on sprint running performance may be because the power absorption in the hip joint is especially important for sprint running [30,31]. In fact, in barbell hip thrust training, the power improvement is more important to maximize the sprint running performance compared to the strength improvement [11]. In sprint running, the hip extension moment is generated during the latter part of the swing phase, and the negative power is produced during the beginning of the latter part to decelerate the forward leg-swing motion [30]. Maximizing the running speed from the middle of the acceleration zone until the maximal speed zone is positively associated with increases in power absorption; however, it is not associated with increases in positive generation [30,31]. Therefore, the barbell hip thrust would be a more useful exercise for athletes to produce a high negative power in the hip joint and to improve sprint running performance potentially. Thus, in the barbell hip thrust, calculating the power absorption and power generation and comparing those in the conventional resistance exercises are required to determine the lower-body extension strength during the eccentric and concentric phases. This will provide useful information for athletes and coaches in strength training. For instance, one resistance exercise can be selected because it is more advantageous for improving the extension moment, power absorption and/or power generation in the hip joint.

Previous kinetic studies limited assessing lumbosacral, hip and knee joint moments in the standing resistance exercises [32–42]; for instance, deadlift is conducted by hip extensor moment, while back squat is conducted by knee extension moment [43]. One possibility of hip or knee domination is likely to be caused by the different joint angles in which the peak joint moments were generated in the lower limb. Different joint-angle strength trainings lead to the different specific joint angle-moment relationships throughout the neuromuscular adaptation [44]. This would conclusively affect the sensitivity of the transformation of strength training into specific dynamic motions.

In the barbell hip thrust, the trunk completely comes in contact with a bench or a box to lift the barbell; so, an external force onto the trunk needs to be measured for the calculation of whole-body kinetics. This suggests that kinetics in the lumbosacral joint, which is directly affected by an external force on the trunk, in the barbell hip thrust is different from that in the standing exercises without the external force on the trunk [45,46]. It has been recently reported that trunk kinetics are related to the high performance in dynamic motions [45,46]. Extension kinetics in the lumbopelvic joint connecting the trunk with the pelvis is an important factor for high performance in sprint running [45] and jumping [46]. Thus, in a barbell hip thrust, kinetic parameters involving the joint angle-moment relationship need to be calculated by considering all external forces and to be compared to those in standing resistance exercises.

The purpose of this study was to determine kinetic characteristics of the lumbosacral, hip and knee joints in the barbell hip thrust, compared to those in representative standing resistance exercises. This information will draw out the effectiveness of the barbell hip thrust compared to deadlift and back squat for better training methodology to maximize dynamic motions such as sprint running. We hypothesized three main hypotheses: First, lumbosacral kinetics in the barbell hip thrust would be different from those in the deadlift and back squat. Second, hip kinetics would be larger in the barbell hip thrust than in the back squat and would be different from those in the deadlift. Third, knee kinetics in the barbell hip thrust would be different from those in the deadlift and back squat.

## Materials and methods

### Participants

Eleven well-trained male sprinters (age, 21.3 ± 1.3 years; body mass, 69.1 ± 7.5 kg; height, 177 ± 5 cm; 100-m personal record, 11.10 ± 0.38 s; training duration, 8.5 ± 2.1 years; all measurements are mean ± standard deviation [SD]) volunteered to participate in this study. The participants trained once a day for approximately 120 min, 5 days per week. The participants had at least one year of experience with strength training for the barbell hip thrust, deadlift and back squat. Written informed consent from the participants was obtained after they received a verbal explanation of the purpose, benefits and potential risks of the study. All procedures were conducted in accordance with the Helsinki Declaration and approved by the Research Ethics Committee involving Living Human Subjects at Ritsumeikan University (BKC-human-2017-001).

### Experimental approach to the problem

Participant performed two full range of motion (ROM) repetitions using their previously determined six-repetition maximum (6-RM) loads, for each of the randomly ordered test exercises so as to compare kinetic parameters in the barbell hip thrust, deadlift and back squat. Six-RM is a load used for strength development in resistance training programs [47].

### Methodology

Participants joined a two-day pre-testing session and one-day primary testing session. Prior to each session, participants performed self-warm up for twenty minutes involving jogging, dynamic stretching and 10 lifts with less than 50% of 1RM. All participants were acquainted with the test procedures during the pre-testing session. On the first day of the pre-testing session, participants were instructed to perform each of the three resistance exercises to determine the 6-RM. Approximately one week after the pre-testing session, 6-RMs were measured and determined in the barbell hip thrust (156.4 ± 14.5 kg), deadlift (117.9 ± 17.0 kg) and back squat (117.7 ± 22.4 kg). On the primary testing session, approximately 72 hours after the second day of the pre-testing session, each participant performed two trials of two full range of motion (ROM) repetitions using their previously determined 6-RM loads, for each of the randomly ordered test exercises. A 5 min recovery period was provided between the trials and exercises to reduce order and fatigue effects [48].

**Fig 1:**
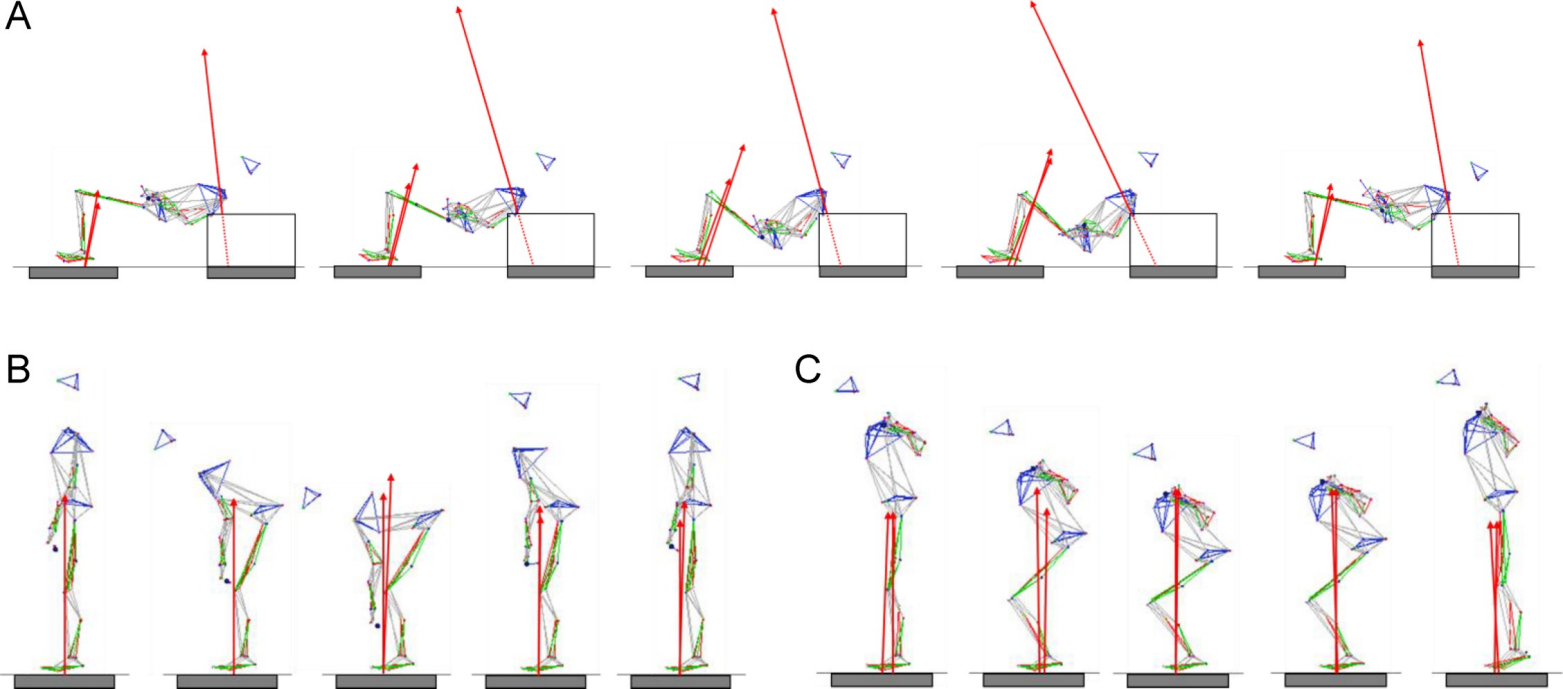
Typical stick postures during the barbell hip thrust (A), deadlift (B) and back squat (C). Red arrows indicate the ground reaction forces captured by force plates (grey areas).

In the barbell hip thrust, the barbell was placed on the pelvis over the hips [6] and the scapulae was on the box (height: 0.36 m). During the descent phase, participants descended by flexing the hips and knees until the weight plates (diameter: 45.0 cm; height of center of the bar: 22.5 cm) reached as close to the ground as possible. During the ascent phase of the exercise, participants lifted the barbell by extending the hips and knees until the torso (the line between the acromion and the anterior superior iliac spine) was parallel to the ground (Fig 1). During both the descent and ascent phases, participants kept the shin vertically to the floor. In the deadlift, participants gripped the barbell firmly by using straps. Participants descended by flexing the hips and knees until the weight barbell reached as close to the ground as possible, keeping the hips lower than the shoulders and elbows fully extended. During the ascent phase of the exercise, participants lifted the barbell by extending the hips and knees. As the barbell was raised as a result of hip and knee extension, participants were instructed to keep the barbell close to the shins and thighs through the ROM of the lift, which was completed when full knee and hip extension was attained. In the back squat, participants were instructed to position the barbell at the base of the neck. Participants descended by flexing the hips and knees until the knee flexion angle is 90° and ascended using the same method. A half-squat depth was selected for the instruction because it has been reported that squats with deeper knee bends do not relate to the sprint performance [49], whereas a 90° knee flexion angle affects it [49–53]. As a common instruction in the three resistance exercises, participants griped the barbell with a closed pronated grip. The participants’ feet were flat on the floor using a shoulder width bilateral stance. Participants were instructed to push or pull “as hard and fast as possible” during the descent and ascent phases. However, participants were also instructed to prevent the barbell from having momentary flight time after the ascent phase, and the participants were not allowed to jump off the ground after the ascent phase.

During the strength exercises, reflective marker position data and ground reaction forces (GRFs) were captured simultaneously. A total of 61 retro-reflective markers sized 12 mm were attached to the whole body and the barbell. The three-dimensional locations of the markers were sampled using 22 high-speed infrared cameras (Raptor-E Digital; Motion Analysis Corporation, Santa Rosa, CA, United States) at 250 Hz. GRFs generated by the two lower limbs were separately measured using two strain-gaged-type force plates (TF-4060-B; Tec Gihan Inc., Kyoto, Japan). During the barbell hip thrust, the box was fixed onto the other two force plates and used the combined GRF as an external force of the trunk segment. The center of pressure (COP) of the combined GRF was moved to the upper surface of the box. The GRF data were sampled at 1000 Hz.

A 15-segment rigid body model, with the head, trunk, pelvis, upper arms, forearms, hands, thighs, shanks and feet was created. Segmental mass data was used from Ae et al.’s values [54]. The moment of inertia and center of gravity location for each segment were calculated using the segment geometry technique [55]. The location of the lumbosacral [56] and hip joints [57] were estimated using a pelvis geometry model and estimated the locations of the neck, elbow, wrist, knee and ankle joints were used as the mid-point between two marker positions attached around the joint. The reflective marker attached on the lateral position of the acromion was used as the shoulder joint. Fourth-order zero-lag low-pass Butterworth filters were used for the marker trajectory data with a cut-off frequency of 3 Hz because of the slow motions and were used for GRF data with optimal cut-off frequencies by residual analysis [58].

Three-dimensional analysis was recruited for a more accurate kinetic calculation rather than two-dimensional analysis [36,37]. Joint angle and angular velocity were three-dimensionally calculated by the standard calculation [58]. So as to calculate the joint moments, we used a motion equation that involved a 15-link rigid human model, which can be expressed as follows:

$$M\dot{t}+Kt-Mg=H_{I}\hat{f}+H_{ext}{\hat{f}}_{ext}$$ (1)

where ***M*** is whole inertia matrix and $t={\left[{\ddot{A}}_{0}\;{\dot{\omega }}_{0}\;{\ddot{A}}_{1}\;{\dot{\omega }}_{1}\cdots {\ddot{A}}_{14}\;{\dot{\omega }}_{14}\right]}^{T}$ is the generalized acceleration vector that consists of the linear acceleration vector ${\ddot{A}}_{i}$ at the proximal end point of segment *i* (*i* = 0,…,14) and the angular acceleration vector ${\dot{\omega }}_{i}$ at segment *i* (all symbols are defined in Table 1). All matrix and vector are described in the world coordinate system. Subscript _*i*_ denotes the segment number (0: pelvis, 1: trunk, 2: head, 3: right upper arm, 4: right forearm, 5: right hand, 6: left upper arm, 7: left forearm, 8: left hand, 9: right thigh, 10: right shank, 11: right foot, 12: left thigh, 13: left shank and 14: left foot). ***Kt*** is Coriolis and centrifugal force vector. ***Mg*** is the gravitational force vector. $H_{I}\hat{f}$ is the term in which joint force and moment applied between the two segments are involved. $\hat{f}={\left[F_{1}\;N_{1}\;F_{2}\;N_{2}\cdots F_{14}\;N_{14}\right]}^{T}$ consists of the joint force vector ***F***_***k***_ and joint moment vector ***N***_***k***_ of joint *k* (*k* = 1,…,14). Subscript _*k*_ denotes the joint number (1: lumbosacral, 2: neck, 3: right shoulder, 4: right elbow, 5: right wrist, 6: left shoulder, 7: left elbow, 8: left wrist, 9: right hip, 10: right knee, 11: right ankle, 12: left hip, 13: left knee and 14: left ankle).$H_{ext}{\hat{f}}_{ext}$ is the term in which external force applied at segment *i* (*i* = 0,1,5,8,11,14) are involved. ${\hat{f}}_{ext}={\left[F_{ext,0}\;N_{ext,0}\;F_{ext,1}\;N_{ext,1}\cdots F_{ext,14}\;N_{ext,14}\right]}^{T}$ consists of GRF vector ***F***_***ext*,*i***_ and free moment vector ***N***_***ext*,*i***_ on the COP of segment *i*. The barbell force was assumed to be the mass and acceleration of the barbell being lifted. Matrices ***M***, ***K***, ***H***_***I***_, and ***H***_***ext***_ are explained in S1 Appendix. Using Eq (1), the dynamic equation to calculate joint forces and moments of all 14 joints can be finally expressed as follows:

$$\hat{f}={\left(H_{I}^{T}H_{I}\right)}^{-1}H_{I}^{T}(M\dot{t}+Kt-Mg-H_{ext}{\hat{f}}_{ext})$$ (2)

**Table 1:** Definition of symbols used in equations of motion.

Variables	Definition
*w*	superscript expressing world coordinate system
***M***	whole inertia matrix
$\dot{t}$	generalized acceleration vector
***K***	Coefficient matrix for Coriolis and centrifugal force
***t***	generalized velocity vector
***g***	gravitational acceleration vector
***H***_***I***_	coefficient matrix for the joint reaction force and moment
$\hat{f}$	joint force and moment vector
***H***_***ext***_	coefficient matrix for the ground reaction force and free moment
${\hat{f}}_{ext}$	external force vector
*i*	subscript expressing segment number
${\ddot{A}}_{i}$	linear acceleration vector of segment *i*
${\dot{\omega }}_{i}$	angular acceleration vector of segment *i*
*k*	subscript expressing joint number
***F***_***k***_	joint force vector of joint *k*
***N***_***k***_	joint moment vector of joint *k*
***F***_***ext*,*i***_	ground reaction force vector of segment *i*
***N***_***ext*,*i***_	free moment vector on the center of pressure of segment *i*

Based on the purpose in this study, we focused on the lumbosacral, hip and knee joint moments of the right leg.

Finally, joint kinematic and kinetic parameters were transformed from the world coordinate system to each proximal segment coordinate system. The pelvis segment was defined as the most proximal segment. The joint power generation/absorption was calculated conventionally [58]. The joint extension moment and power generation are always written in positive values and joint flexion moment and power absorption are always written in negative values. Two trials were conducted in each resistance exercise, so the kinetic parameters of four total full ROM repetitions were averaged for each participant for further analyses. All analyses were performed using Matlab version 9.3.0.713579 (The Mathworks, Natick, MA, USA).

### Statistical analysis

Data are given as group mean values ± SD. After normality of variances of the distributions was checked by the Lilliefors test and we confirmed that all variances normally distributed. Differences of peak values of kinetic parameters were evaluated among the barbell hip thrust, deadlift and back squat using a repeated analysis of variance (ANOVA). Bonferroni pairwise comparisons were performed as a post hoc analysis if significant differences were found (*P* < 0.05). Relative reliabilities of the kinetic parameters within-participant variability across the four repetitions were determined using intraclass correlation coefficient (ICC). For the calculation of ICCs, two-way mixed-effects model and the absolute agreement was selected [59]. Interpretation was as follows: <0.49, poor; between 0.50 and 0.74, moderate; between 0.75 and 0.89, good; > 0.90, excellent [59]. The effect sizes (ESs) were calculated using Cohen’s *d* (See S1 Table and S1 Fig in detail).

## Results

**Fig 2:**
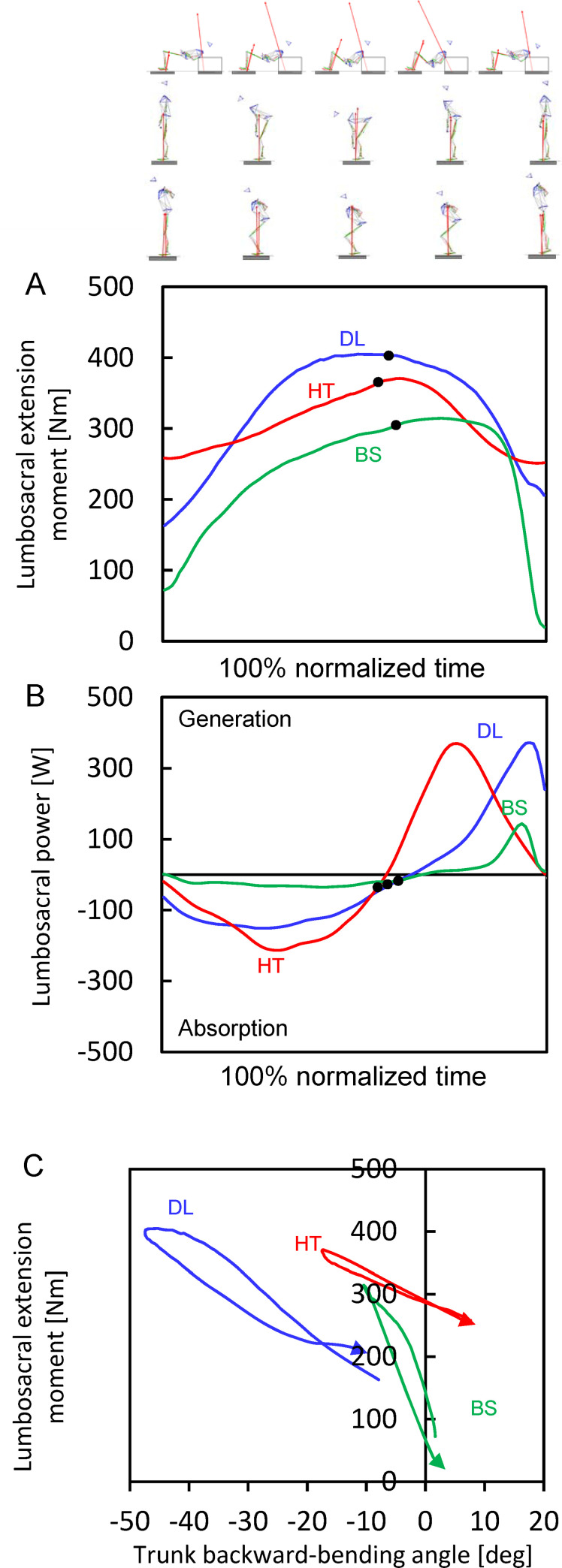
Mean values of the time-series lumbosacral-joint kinetic parameters of all participants. (A) Joint moment. (B) Power. (C) Joint angle-moment relationship. Red, blue and green lines indicate the mean values in the barbell hip thrust (HT), deadlift (DL) and back squat (BS). Descent and ascent phases are divided by a black filled circle in each exercise curve.

**Fig 3:**
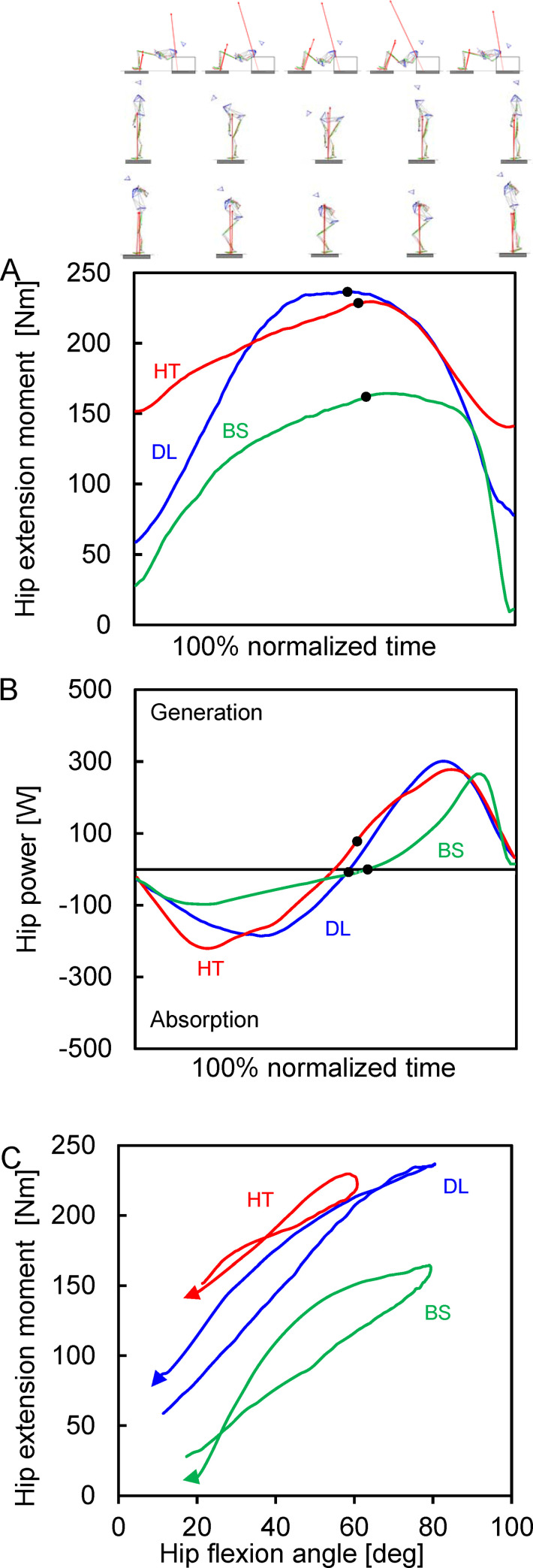
Mean values of the time-series hip-joint kinetic parameters of all participants. (A) Joint moment. (B) Power. (C) Joint angle-moment relationship. Red, blue and green lines indicate the mean values in the barbell hip thrust (HT), deadlift (DL) and back squat (BS). Descent and ascent phases are divided by a black filled circle in each exercise curve.

**Fig 4:**
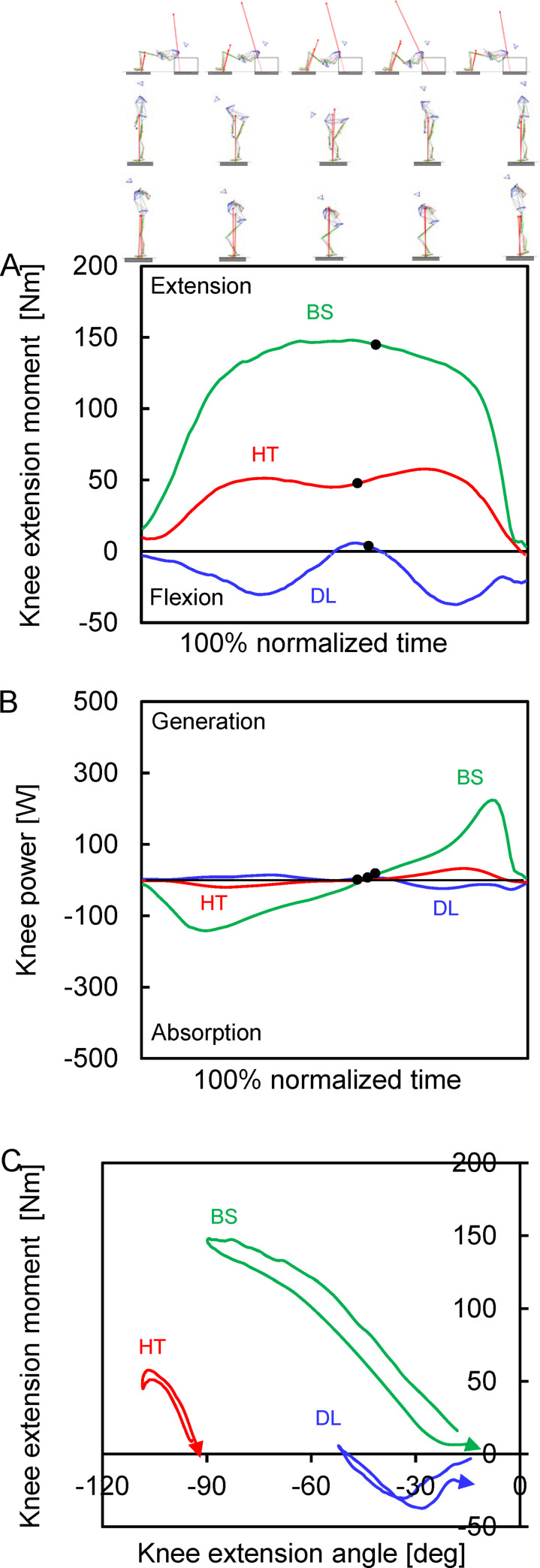
Mean values of the time-series knee-joint kinetic parameters of all participants. (A) Joint moment. (B) Power. (C) Joint angle-moment relationship. Red, blue and green lines indicate the mean values in the barbell hip thrust (HT), deadlift (DL) and back squat (BS). Descent and ascent phases are divided by a black filled circle in each exercise curve.

In the lumbosacral and hip joints, the extension moment was generated throughout the entire descent and ascent phases in the barbell hip thrust, deadlift and back squat and the peak values were generated at almost last instant during the descent phase/initial instant during the ascent phase (Figs 2A and 3A). The peak values of lumbosacral and hip extension moments were significantly larger in the barbell hip thrust than those in the back squat (differences: 24 ± 21% and 42 ± 24%); however, no significant differences were observed in those between the barbell hip thrust and deadlift (Table 2; raw data behind group mean values ± SD for Table 2 are shown in S1 Table). In the lumbosacral and hip joints in three different exercises, the power absorption and generation occurred during the descent and subsequent ascent phases, respectively (Figs 2B and 3B). The peak values of power absorption in the lumbosacral and hip joints were significantly larger in the barbell hip thrust than those in the back squat. In the barbell hip thrust, larger peak power generation was observed in the lumbosacral joint than that in the back squat. No significant difference was observed in the peak hip power generation between the three different resistance exercises. The ESs are shown in S1 and S2 Figs. All time-series data used to build for Figs 2–4 are shown in S2 Table.

**Table 2:** Comparison of peak values of kinetic parameters among barbell hip thrust (HT), deadlift (DL) and back squat (BS).

	Value of kinetic parameters (mean ± SD)			*P* value of multiple comparisons		
	HT	DL	BS	HT vs DL	HT vs BS	DL vs BS
**Peak extension moment [Nm]**						
** Lumbo-pelvic joint**	385 ± 53	410 ± 67	320 ± 75	0.193	0.004	0.001
** Hip joint**	237 ± 26	242 ± 35	172 ± 37	1.650	< 0.001	< 0.001
** Knee joint**	66 ± 33	24 ± 27	155 ± 28	0.014	< 0.001	< 0.001
**Peak power absorption [W]**						
** Lumbo-pelvic joint**	−270 ± 150	−184 ± 53	−58 ± 28	0.254	0.001	< 0.001
** Hip joint**	−245 ± 87	−204 ± 70	−105 ± 40	0.635	< 0.001	< 0.001
** Knee joint**	−29 ± 14	−40 ± 20	−161 ± 89	0.621	0.002	0.003
** Peak power generation [W]**						
** Lumbo-pelvic joint**	429 ± 290	447 ± 191	166 ± 66	2.417	< 0.001	0.014
** Hip joint**	321 ± 106	349 ± 70	297 ± 84	1.157	0.467	0.270
** Knee joint**	38 ± 29	25 ± 9	273 ± 105	0.593	< 0.001	< 0.001

Significant difference between exercises are highlighted by grey shading.

The knee extension moment was generated during the descent and ascent phases in the barbell hip thrust and back squat; in contrast, the flexion moment was almost generated during both phases in the deadlift (Fig 4A). Therefore, the knee power generation was initially generated during the descent phase in the deadlift; in contrast, the power absorption was initially generated during the descent phase in the barbell hip thrust and back squat (Fig 4B). The peak knee extension moment was significantly smaller in the barbell hip thrust than that in the back squat and was significantly larger than that in the deadlift. Both peak power absorption and generation in the knee joint were significantly smaller in the barbell hip thrust and deadlift than those in the back squat.

Joint moment-angle curves of the lumbosacral, hip and knee joints were different among the barbell hip thrust, deadlift and back squat (Figs 2C, 3C and 4C). The trunk backward-bending angle at the peak extension moment was −18 ± 15° in the barbell hip thrust and was significantly larger (less negative and more forward bend) than in the deadlift (−45 ± 11°, *P* < 0.001) but comparable to the back squat (−11 ± 11°, *P* = 0.477). Hip flexion angle at the peak extension moment was 59 ± 13° in the barbell hip thrust and was significantly less than in the deadlift (78 ± 9°, *P* = 0.003) and back squat (74 ± 8°, *P* = 0.018). Knee extension angle at the peak extension moment was −106 ± 11° in the barbell hip thrust and was significantly less than in the deadlift (−43 ± 18°, *P* < 0.001) and back squat (−85 ± 7°, *P* < 0.001).

The minimum value of ICC was 0.76 of the peak power generation of the knee joint in the deadlift and can be classified as being good. ICCs of the other kinetic parameters were 0.86–1.00 in the barbell hip thrust and back squat. Therefore, the relative reliability of all data can be classified as good or excellent.

## Discussion

Our main findings were as follows: Firstly, in the lumbosacral and hip joints, the peak extension moments were larger in the barbell hip thrust and deadlift compared to those in the back squat. Similar tendencies were observed in the peak power absorption and generation, which occurred during the descent and ascent phases, respectively. Secondly, in the knee joint, the largest was the peak extension moment in the back squat, followed in order by that in the barbell hip thrust and that in the deadlift. The peak knee negative and positive extension powers were clearly larger in the back squat rather than those in the barbell hip thrust and deadlift. These may demonstrate that a barbell hip thrust can be a lower back and hip extension exercise for strength training, accompanied by a slight knee extension.

Few previous studies calculated the lumbosacral, hip and knee joint moments during the resistance exercises [32–42]. It is not easy to discuss whether our calculated value of the joint moment is valid, compared to that in previous studies on the resistance exercise. This is because the calculated value changes by differences of barbell load [16]. In contrast in this study, we used same relative resistance load in the different resistance exercises. Moreover, the relative reliability of all kinetic parameters was classified as good or excellent. Therefore, when discussing the kinetics of the three resistance exercises herein, we avoid comparisons of kinetics by our own calculation against those from previous studies on resistance exercises.

With regard to the lumbosacral kinetics, in dynamic motion with a maximal effort such as sprint running, the peak lumbosacral extension moment normalized by the body mass was 2.89 ± 0.64 Nm/kg [45] and the estimated absolute value is 185 Nm. The peak lumbosacral extension moment in the barbell hip thrust was 385 ± 53 Nm and was almost two times larger than that during the sprint running [45]. This suggests that a barbell hip thrust can be regarded as a resistance exercise strengthening the lower back extension so as to maximize the dynamic motion performance potentially.

The peak lumbosacral extension moment in the barbell hip thrust was similar to 410 ± 67 Nm of that in the deadlift, demonstrating that our first hypothesis was partially not accepted. In contrast, the trunk forward-bending angle at the peak extension moment in the deadlift was approximately 2.5 times as large as that in the barbell hip thrust (−45 ± 11° vs −18 ± 15°). These flexion angles in the deadlift and barbell hip thrust were almost the same as those during the acceleration zone and maximal speed zone, respectively, for sprinters [60]. Different joint-angle strength trainings lead to the different specific joint angle-moment relationships throughout the neuromuscular adaptation [44]. This might indicate that, as the practical application and from the perspective of lumbosacral joint, deadlift and barbell hip thrust can be used separately according to their intended purposes, enabling transformations of strength training into accelerated or maximal-speed runs for sprinters.

The joint angle affects the sarcomere length of the muscle, changing the muscle force [12], and finally changes the joint moment. Moreover, the joint angle also affects the moment arm and ultimately changes the joint moment. Therefore, the extension force production is required to be discussed considering the joint moment-angle relationship. A larger hip extension moment is generated at a more extended (closer to full extension) position in the knee joint [61]; in contrast, the knee flexion-extension motion in the barbell hip thrust was conducted at a more flexed knee position than in the deadlift (Fig 3C; knee extension angle at the peak extension moment: −43 ± 18° vs −106 ± 11°). Furthermore, one EMG study suggests that a hip extension moment seems to be weaker in the barbell hip thrust than that in the deadlift because activation in only biceps femoris was lower in the hip extensor muscles [5]. Nevertheless, in this study, the peak hip extension moment in the barbell hip thrust was not significantly smaller than that in the deadlift. Based on a previous study, the preferred hip flexion position (53°) to generate a large hip extension moment can be observed using a mathematical model [62]. Therefore, the smaller hip flexion angle (closer to 53°) for yielding the maximum hip extension moment in the barbell hip thrust (59 ± 13°) is more desirable when compared to the deadlift (78 ± 9°) (Fig 3C).

Sprint performance is significantly related to 1RM loads in the squats with the shallower knee bends [44]. This might be owing to the joint-angle specificity in sprint running [63]: the peak hip flexion angles during the squats with shallower knee bends are closer to that during sprint running (approximately 60–85° by Ito et al. [64]). Similarly, the hip flexion angles at the peak hip extension moment were 59 ± 13°, 78 ± 9° and 74 ± 8° in the barbell hip thrust, deadlift and back squat, respectively. However, the hip power absorption, which is more important for enhancing dynamic motion performance [30,31], was larger in the barbell hip thrust and deadlift compared to that in the squat. These suggest that sprint running performance can be enhanced effectively by improved hip power absorption by resistance training involving a barbell hip thrust and deadlift rather than a half squat. Thus, our second hypothesis was partially accepted.

With regard to the knee extension moment, the peak value in the barbell hip thrust was approximately three times larger than that in the deadlift. This can be regarded as a main kinetic difference between the barbell hip thrust and deadlift. The difference was caused by the fact that in the deadlift, the flexion moment was generated in the knee joint almost throughout the entire descent and ascent phases, which was not observed in the barbell hip thrust. This knee flexion force production in the deadlift was also observed in a previous study [17,32,36]. Therefore, the power production was conversely observed in the descent phase in the deadlift. In contrast, the knee extension kinetics were clearly smaller in the barbell hip thrust than those in the back squat. This may be because the knee motion in the barbell hip thrust was conducted at a more flexed position compared to that in the back squat. As the knee extension angle was always less than −90° in the barbell hip thrust, this may lead to a weaker knee extension moment because of the longer sarcomere from the optimal length [62]. Interestingly, previous studies reported that the EMG activity of the vastus lateralis muscle, the knee extensor muscle, was similar in the barbell hip thrust to that in the back squat [6,65] and deadlift [66] and did not support our finding. Therefore, it is unclear what exactly caused the lower knee extension moment in the barbell hip thrust in our study. Further research is required to investigate the reasons for knee extension/flexion kinetic difference among the barbell hip thrust, deadlift, and back squat. Thus, our third hypothesis was accepted.

The stretch-shortening cycle, which is characterized by the enhancement of power production in a concentric muscle action preceded by active muscle lengthening, can be observed during both dynamic motion [23–29] and resistance exercise [16]. In the barbell hip thrust, in the lumbosacral, hip and knee joints, the eccentric and concentric motions occurred during the descent and ascent phases, respectively. Unfortunately, no significant difference in the peak value of hip power generation was observed between the barbell hip thrust and back squat. The reason may be that the small sample size led to no significant difference (see, S2 Fig panel C in S1 Fig). However, some previous studies reported that increases in sprinting speed is not affected by increases in power generation in the hip joint but instead by the increases in power absorption in the hip during the swing phase [30,31]. Therefore, the insignificant peak power generation may not be the most critical determinant in the exercise selection of strength training for improving sprint running performance.

We have three limitations in this study. First, in this study, only one barbell load (6-RM) was used for testing. The power output under several loads changed based on the resistance exercise. Further research is needed to determine the force–velocity and force–power relationships and to determine the highest power generation under several barbell loads in the barbell hip thrust compared to the deadlift in the future. Second, the range of motion was restricted by the bench height and barbell diameter of the barbell hip thrust. The box height was 0.36 m; therefore, several participants could not lead to the hip neutral position or the hyperextension when the torso was parallel to the ground at the end of the ascent phase. The barbell diameter restricted the maximal hip flexion angle in the barbell hip thrust compared to that in the squat and deadlift. The size of training equipment should be individually normalized by the participant body size (for example, shin length) for the same range of motion. Third, in each resistance exercise, only one exercise type was selected, and several exercise variations could not be set. The joint kinetic parameters change based on the exercise variations; for instance, there are different EMG amplitudes among barbell, band, and American hip thrusts (7). Further research is required to calculate kinetic parameters under different ranges of motion and/or under different exercise variations in each resistance exercise and discuss their transformations of strength training into specific dynamic motions.

## Conclusions

In conclusion, this study found that in the barbell hip thrust, as well as deadlift, the peak values of the lumbosacral and hip extension moments were 24% and 42% larger than those in the back squat, respectively. These demonstrated that a barbell hip thrust, as well as deadlift, can be a useful resistance exercise to strengthen the lower back and posterior thigh muscles. The trunk forward-bending angle at the peak extension moment was smaller in the barbell hip thrust compared to that in the deadlift. This might indicate that, as the practical application, deadlift and barbell hip thrust can be used separately according to their intended purposes, enabling transformations of strength training into accelerated or maximal-speed runs for sprinters.

## Supporting information

S1 Fig Effect sizes (Cohen’s *d* ± 95% confidence limit) in the kinetic parameters between the barbell hip thrust, deadlift and back squat. (PDF)

S2 Fig Effect sizes (Cohen’s *d* ± 95% confidence limit) in the joint angle at the peak extension moment between the barbell hip thrust, deadlift and back squat. (PDF)

S1 Table Raw data behind mean values ± SD for Table 2. (XLSX)

S2 Table All time-series data used to build for Figs 2–4. (XLSX)

S1 Appendix (PDF)
