# Supplementary material for: Kinetics in lumbosacral and lower-limb joints of sprinters during barbell hip thrust compared to deadlift and back squat
Source: PLoS One. 2021 Jul 1;16(7):e0251418. doi: 10.1371/journal.pone.0251418 (PMC8248606; doi:10.1371/journal.pone.0251418)
Supplement: S2 Fig — (PDF) [file pone.0251418.s002.pdf]

Supporting Information Otsuka et al. (2021)

S4. Effect sizes (Cohen's *d* ± 95% confidence limit) in the joint angle at the peak extension moment between the barbell hip thrust, deadlift and back squat.

Effect sizes (ESs) were calculated using Cohen's *d* and the interpretation of |ES| was as follows: < 0.19, trivial; between 0.20 and 0.49, small; between 0.5 and 0.79, medium; > 0.80, large [1]. Ninety-five percent (95%) confidence limits (CLs) of ES were calculated for magnitude-based inferences [2].

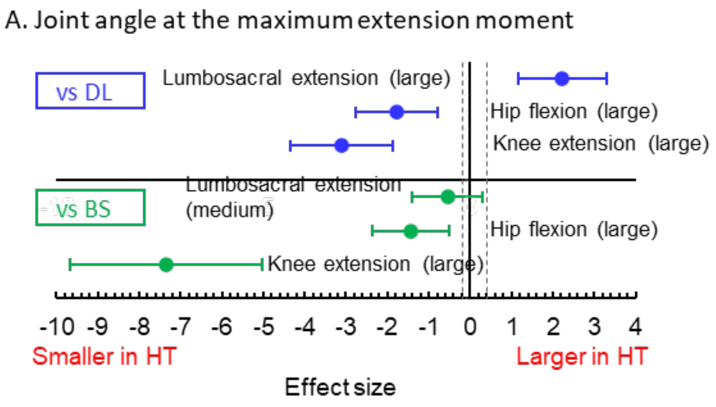

S3 Fig. Effect sizes (Cohen's *d* ± 95% confidence limit) in the joint angle at the peak extension moment between the barbell hip thrust [HT], deadlift [DL] and back squat [BS]. The area ± 0.2 from zero in the middle demonstrates the small effect. The interpretations were based on the Hopkins et al.'s previous study [2].

References

1. Cohen J. Statistical Power Analysis for the Behavioral Sciences. New York, NY: Routledge Academic; 1988.
2. Hopkins WG, Marshall SW, Batterham AM, Hanin J. Progressive statistics for studies in sports medicine and exercise science. Med Sci Sports Exerc. 2009; 41:3-13. doi: 10.1249/MSS.0b013e31818cb278
