# Supplementary material for: Kinetics in lumbosacral and lower-limb joints of sprinters during barbell hip thrust compared to deadlift and back squat
Source: PLoS One. 2021 Jul 1;16(7):e0251418. doi: 10.1371/journal.pone.0251418 (PMC8248606; doi:10.1371/journal.pone.0251418)
Supplement: S1 Appendix — (PDF) [file pone.0251418.s005.pdf]

Supporting Information Otsuka et al. (2021)

S1 Appendix

Whole inertia matrix ${}^{w}\boldsymbol{M}$ is described as

$$ {}^{w}\boldsymbol{M} = \begin{bmatrix} {}^{w}\boldsymbol{M_0} & \boldsymbol{0} & \cdots & \boldsymbol{0} \\ \boldsymbol{0} & {}^{w}\boldsymbol{M_1} & \cdots & \boldsymbol{0} \\ \vdots & \vdots & \ddots & \vdots \\ \boldsymbol{0} & \boldsymbol{0} & \cdots & {}^{w}\boldsymbol{M_{14}} \end{bmatrix} \tag{1'} $$

$$ {}^{w}\boldsymbol{M_i} = \begin{bmatrix} m_i\boldsymbol{I} & -m_i[{}^{w}\boldsymbol{r_i}\times] \\ m_i[{}^{w}\boldsymbol{r_i}\times] & {}^{w}\boldsymbol{I_i} - m_i[{}^{w}\boldsymbol{r_i}\times]^2 \end{bmatrix} \tag{2'} $$

where ${}^{w}\boldsymbol{M_i}$ is segmental inertia matrix involving a skew symmetric matrix $[{}^{w}\boldsymbol{r_i}\times]$. ${}^{w}\boldsymbol{r_i}$ is the vector from a proximal end to the centre of mass of segment *i* in world coordinate system. $\boldsymbol{I}$ is unit matrix, m_i is the mass of segment *i* and ${}^{w}\boldsymbol{I_i}$ is the inertial moment of segment *i*. ${}^{w}\boldsymbol{K}$ with respect to Coriolis and centrifugal force vector is described as

$$ {}^{w}\boldsymbol{K} = \begin{bmatrix} {}^{w}\boldsymbol{K_0} & \boldsymbol{0} & \cdots & \boldsymbol{0} \\ \boldsymbol{0} & {}^{w}\boldsymbol{K_1} & \cdots & \boldsymbol{0} \\ \vdots & \vdots & \ddots & \vdots \\ \boldsymbol{0} & \boldsymbol{0} & \cdots & {}^{w}\boldsymbol{K_{14}} \end{bmatrix} \tag{3'} $$

$$ {}^{w}\boldsymbol{K_i} = \begin{bmatrix} \boldsymbol{0} & -m_i[{}^{w}\boldsymbol{\omega_i}\times][{}^{w}\boldsymbol{r_i}\times] \\ \boldsymbol{0} & [{}^{w}\boldsymbol{\omega_i}\times]{}^{w}\boldsymbol{I_i} - m_i[{}^{w}\boldsymbol{r_i}\times][{}^{w}\boldsymbol{\omega_i}\times][{}^{w}\boldsymbol{r_i}\times] \end{bmatrix} \tag{4'} $$

${}^{w}\boldsymbol{H}_{I}$ is described as

$$ {}^{w}\boldsymbol{H}_{I} = \begin{bmatrix} \mathbf{0}_{6*84} \\ \boldsymbol{I}_{84*84} \end{bmatrix} - {}^{w}\boldsymbol{H} \qquad (\mathbf{5}') $$

where

$$^{w}\boldsymbol{H} = \begin{bmatrix}
^{w}\boldsymbol{h}_{0,1} & \mathbf{0} & \mathbf{0} & \mathbf{0} & \mathbf{0} & \mathbf{0} & \mathbf{0} & \mathbf{0} & ^{w}\boldsymbol{h}_{0,9} & \mathbf{0} & \mathbf{0} & ^{w}\boldsymbol{h}_{0,12} & \mathbf{0} & \mathbf{0} \\
\mathbf{0} & ^{w}\boldsymbol{h}_{1,2} & ^{w}\boldsymbol{h}_{1,3} & \mathbf{0} & \mathbf{0} & ^{w}\boldsymbol{h}_{1,6} & \mathbf{0} & \mathbf{0} & \mathbf{0} & \mathbf{0} & \mathbf{0} & \mathbf{0} & \mathbf{0} & \mathbf{0} \\
\mathbf{0} & \mathbf{0} & \mathbf{0} & \mathbf{0} & \mathbf{0} & \mathbf{0} & \mathbf{0} & \mathbf{0} & \mathbf{0} & \mathbf{0} & \mathbf{0} & \mathbf{0} & \mathbf{0} & \mathbf{0} \\
\mathbf{0} & \mathbf{0} & \mathbf{0} & ^{w}\boldsymbol{h}_{3,4} & \mathbf{0} & \mathbf{0} & \mathbf{0} & \mathbf{0} & \mathbf{0} & \mathbf{0} & \mathbf{0} & \mathbf{0} & \mathbf{0} & \mathbf{0} \\
\mathbf{0} & \mathbf{0} & \mathbf{0} & \mathbf{0} & ^{w}\boldsymbol{h}_{4,5} & \mathbf{0} & \mathbf{0} & \mathbf{0} & \mathbf{0} & \mathbf{0} & \mathbf{0} & \mathbf{0} & \mathbf{0} & \mathbf{0} \\
\mathbf{0} & \mathbf{0} & \mathbf{0} & \mathbf{0} & \mathbf{0} & \mathbf{0} & \mathbf{0} & \mathbf{0} & \mathbf{0} & \mathbf{0} & \mathbf{0} & \mathbf{0} & \mathbf{0} & \mathbf{0} \\
\mathbf{0} & \mathbf{0} & \mathbf{0} & \mathbf{0} & \mathbf{0} & \mathbf{0} & ^{w}\boldsymbol{h}_{6,7} & \mathbf{0} & \mathbf{0} & \mathbf{0} & \mathbf{0} & \mathbf{0} & \mathbf{0} & \mathbf{0} \\
\mathbf{0} & \mathbf{0} & \mathbf{0} & \mathbf{0} & \mathbf{0} & \mathbf{0} & \mathbf{0} & ^{w}\boldsymbol{h}_{7,8} & \mathbf{0} & \mathbf{0} & \mathbf{0} & \mathbf{0} & \mathbf{0} & \mathbf{0} \\
\mathbf{0} & \mathbf{0} & \mathbf{0} & \mathbf{0} & \mathbf{0} & \mathbf{0} & \mathbf{0} & \mathbf{0} & \mathbf{0} & \mathbf{0} & \mathbf{0} & \mathbf{0} & \mathbf{0} & \mathbf{0} \\
\mathbf{0} & \mathbf{0} & \mathbf{0} & \mathbf{0} & \mathbf{0} & \mathbf{0} & \mathbf{0} & \mathbf{0} & \mathbf{0} & ^{w}\boldsymbol{h}_{9.10} & \mathbf{0} & \mathbf{0} & \mathbf{0} & \mathbf{0} \\
\mathbf{0} & \mathbf{0} & \mathbf{0} & \mathbf{0} & \mathbf{0} & \mathbf{0} & \mathbf{0} & \mathbf{0} & \mathbf{0} & \mathbf{0} & ^{w}\boldsymbol{h}_{10,11} & \mathbf{0} & \mathbf{0} & \mathbf{0} \\
\mathbf{0} & \mathbf{0} & \mathbf{0} & \mathbf{0} & \mathbf{0} & \mathbf{0} & \mathbf{0} & \mathbf{0} & \mathbf{0} & \mathbf{0} & \mathbf{0} & \mathbf{0} & \mathbf{0} & \mathbf{0} \\
\mathbf{0} & \mathbf{0} & \mathbf{0} & \mathbf{0} & \mathbf{0} & \mathbf{0} & \mathbf{0} & \mathbf{0} & \mathbf{0} & \mathbf{0} & \mathbf{0} & \mathbf{0} & ^{w}\boldsymbol{h}_{12,13} & \mathbf{0} \\
\mathbf{0} & \mathbf{0} & \mathbf{0} & \mathbf{0} & \mathbf{0} & \mathbf{0} & \mathbf{0} & \mathbf{0} & \mathbf{0} & \mathbf{0} & \mathbf{0} & \mathbf{0} & \mathbf{0} & ^{w}\boldsymbol{h}_{13,14} \\
\mathbf{0} & \mathbf{0} & \mathbf{0} & \mathbf{0} & \mathbf{0} & \mathbf{0} & \mathbf{0} & \mathbf{0} & \mathbf{0} & \mathbf{0} & \mathbf{0} & \mathbf{0} & \mathbf{0} & \mathbf{0}
\end{bmatrix} \quad (\mathbf{6'})$$

$$ {}^{w}\boldsymbol{h}_{i,j} = \begin{bmatrix} \boldsymbol{I} & \boldsymbol{0} \\ [{}^{w}\boldsymbol{p}_{i,j} \times] & \boldsymbol{I} \end{bmatrix} \qquad (7') $$

where ${}^{w}\boldsymbol{h}_{i,j}$ is the matrix involving a skew symmetric matrix $[{}^{w}\boldsymbol{p}_{i,j} \times]$. ${}^{w}\boldsymbol{p}_{i,j}$ is the vector from a proximal end position of segment *i* to that of segment *j* in the world coordinate system.

${}^{w}\boldsymbol{H}_{ext}$ is described as

$$ {}^{w}\boldsymbol{H}_{ext} = \begin{bmatrix} {}^{w}\boldsymbol{H}_{ext,0} & \boldsymbol{0} & \boldsymbol{0} & \boldsymbol{0} & \boldsymbol{0} & \boldsymbol{0} \\ \boldsymbol{0} & {}^{w}\boldsymbol{H}_{ext,1} & \boldsymbol{0} & \boldsymbol{0} & \boldsymbol{0} & \boldsymbol{0} \\ \boldsymbol{0} & \boldsymbol{0} & \boldsymbol{0} & \boldsymbol{0} & \boldsymbol{0} & \boldsymbol{0} \\ \boldsymbol{0} & \boldsymbol{0} & \boldsymbol{0} & \boldsymbol{0} & \boldsymbol{0} & \boldsymbol{0} \\ \boldsymbol{0} & \boldsymbol{0} & \boldsymbol{0} & \boldsymbol{0} & \boldsymbol{0} & \boldsymbol{0} \\ \boldsymbol{0} & \boldsymbol{0} & {}^{w}\boldsymbol{H}_{ext,5} & \boldsymbol{0} & \boldsymbol{0} & \boldsymbol{0} \\ \boldsymbol{0} & \boldsymbol{0} & \boldsymbol{0} & \boldsymbol{0} & \boldsymbol{0} & \boldsymbol{0} \\ \boldsymbol{0} & \boldsymbol{0} & \boldsymbol{0} & \boldsymbol{0} & \boldsymbol{0} & \boldsymbol{0} \\ \boldsymbol{0} & \boldsymbol{0} & \boldsymbol{0} & {}^{w}\boldsymbol{H}_{ext,8} & \boldsymbol{0} & \boldsymbol{0} \\ \boldsymbol{0} & \boldsymbol{0} & \boldsymbol{0} & \boldsymbol{0} & \boldsymbol{0} & \boldsymbol{0} \\ \boldsymbol{0} & \boldsymbol{0} & \boldsymbol{0} & \boldsymbol{0} & \boldsymbol{0} & \boldsymbol{0} \\ \boldsymbol{0} & \boldsymbol{0} & \boldsymbol{0} & \boldsymbol{0} & {}^{w}\boldsymbol{H}_{ext,11} & \boldsymbol{0} \\ \boldsymbol{0} & \boldsymbol{0} & \boldsymbol{0} & \boldsymbol{0} & \boldsymbol{0} & \boldsymbol{0} \\ \boldsymbol{0} & \boldsymbol{0} & \boldsymbol{0} & \boldsymbol{0} & \boldsymbol{0} & \boldsymbol{0} \\ \boldsymbol{0} & \boldsymbol{0} & \boldsymbol{0} & \boldsymbol{0} & \boldsymbol{0} & {}^{w}\boldsymbol{H}_{ext,14} \end{bmatrix} \qquad (8') $$

$$ {}^{w}\boldsymbol{H}_{ext,i} = \begin{bmatrix} \boldsymbol{I} & \boldsymbol{0} \\ [({}^{w}\boldsymbol{A}_{ext,i} - {}^{w}\boldsymbol{A}_{i}) \times] & \boldsymbol{I} \end{bmatrix} \qquad (9') $$

where ${}^{w}\boldsymbol{H}_{\boldsymbol{ext,i}}$ is the coefficient matrix involving a skew symmetric matrix $[({}^{w}\boldsymbol{A}_{\boldsymbol{ext,i}} - {}^{w}\boldsymbol{A}_{\boldsymbol{i}}) \times]$. ${}^{w}\boldsymbol{A}_{\boldsymbol{ext,i}}$ denotes the position of the COP on the segment *i* and ${}^{w}\boldsymbol{A}_{\boldsymbol{i}}$ denotes the position of the proximal end of the segment *i* in the world coordinate system.
